# To the Editors of the Medical and Physical Journal

**Published:** 1810-09

**Authors:** J. A. Paris

**Affiliations:** St. James Street


### *To the Editors of the Medical and Physical Journal.*

GENTLEMEN,

THE Memoir on the Physiology of the Egg, which was read before the Linnean Society, has, I perceive, been honoured with your notice and approbation in the last Number of your Journal, in which you promise to resume the consideration of it in your next. Although I feel proud on the occasion, I must request that you will postpone any farther mention of it until its publication in the Linnean Transactions, before which period, it is irregular to introduce it to public notice.

You are perfectly at liberty to insert this letter, which will explain to your readers, the motive which induces you to abandon the subject you promised to continue.

I am, &c.

J. A. PARIS.

*St. James Street, Aug.* 11, 1810.

